# Evaluation of ATP12A and NFKBIZ as potential markers of inflammatory status in cystic fibrosis airway epithelial cells

**DOI:** 10.1007/s00011-026-02210-z

**Published:** 2026-03-17

**Authors:** Caterina Allegretta, Daniela Guidone, Silvia Boscia, Laura Pisano, Silvia Ricci, Chiara Azzari, Cristina Fevola, Martina De Santis, Fabiana Ciciriello, Enza Montemitro, Daniela Dolce, Giulio Cabrini, Luis J. V. Galietta, Vito Terlizzi, Onofrio Laselva

**Affiliations:** 1 Department of Clinical and Experimental Medicine, University of Foggia, Foggia, Italy; 2 Telethon Institute of Genetics and Medicine, Pozzuoli, Italy; 3 Immunology Unit, Meyer Children’s Hospital IRCCS, Florence, Italy; 4 Department of Health Sciences, University of Florence, Florence, Italy; 5 Department of Pediatric Medicine, Cystic Fibrosis Regional Reference Center, Meyer Children’s Hospital IRCCS, Florence, Italy; 6 Cystic Fibrosis Center, Specialistic Pediatrics Department, Bambino Gesù Children’s Hospital, IRCCS, 00165 Rome, Italy; 7 Department of Life Sciences and Biotechnology, University of Ferrara, Ferrara, Italy; 8 Department of Translational Medical Sciences, University of Napoli ‘Federico II’, Naples, Italy; 9 University of Foggia, Via L. Pinto 1, Foggia, Italy

**Keywords:** Cystic Fibrosis, NFKBIZ, ATP12A, Infection, Inflammation

## Abstract

**Background:**

People with Cystic Fibrosis (pwCF) are prone to bacterial lung infections with *P. aeruginosa*, which have been linked to chronic inflammation in the lung. Although the highly effective CFTR modulator therapy (Elexacaftor-Tezacaftor-Ivacaftor, ETI) has dramatically improved respiratory outcomes in pwCF, airway inflammation and bacterial colonization persist in the upper and lower respiratory tracts.

**Methods:**

We investigated the effect of ETI in both plasma and fresh primary nasal epithelial (HNE) cells obtained from pwCF pre- and post-three months of ETI treatment. Given that inflammation has been shown to upregulate NFKBIZ and the ATP12A proton pump, we measured their levels in fresh HNE cells and in cultured HNE cells exposed to clinical exoproducts (EXO) of *P. aeruginosa* or other inflammatory stimuli.

**Results:**

ELISA analysis revealed a significant reduction of IL-6, IL-8, and IL-17C in both plasma and HNE cells after ETI treatment. NFKBIZ and ATP12A expression was increased after infection and inflammatory stimuli in CF bronchial epithelial (CFBE) and HNE cells, and this increase was reduced by Dimethyl-Fumarate, an anti-inflammatory drug.

**Conclusions:**

These preclinical studies, using patient-derived tissues, suggest that NFKBIZ and ATP12A may play a relevant role in the pathophysiology and inflammatory response of the CF airway epithelium.

**Supplementary Information:**

The online version contains supplementary material available at 10.1007/s00011-026-02210-z.

## Introduction

Cystic Fibrosis (CF) is a genetic, multiorgan, life-threatening disease caused by mutations in the *Cystic Fibrosis Transmembrane Conductance Regulator* (CFTR) gene, which results in viscous secretions accumulating on the surface of epithelial cells, particularly in the lungs and the gastrointestinal tract [1]. The most common CF-causing mutation, the deletion of the amino acid phenylalanine at position 508 (p.Phe508del), affects approximately 90% of people with CF (pwCF). The CFTR modulator (CFTRm) Kaftrio, which combines the potentiator Ivacaftor with two correctors, Elexacaftor and Tezacaftor (ETI), has been already widely administered to patients pwCF with at least one p.Phe508del allele [2]. Although phase 3 clinical trials have demonstrated significant improvement in lung function and a reduction in pulmonary exacerbations with ETI, the overall clinical benefit presents a large inter-individual variability [2, 3]. Even though a decrease in both airway and systemic inflammatory markers within 3–12 months has been confirmed after ETI treatment, the levels of these biomarkers do not fully return to those observed in healthy individuals [4]. Impaired mucociliary clearance is widely recognized as a key factor in establishing a permissive environment for pulmonary infections in pwCF, particularly by opportunistic pathogens like *Pseudomonas aeruginosa (Pa)* [5]. These infections trigger an exaggerated inflammatory response, predominantly driven by activated neutrophils that generate high levels of reactive oxygen species (ROS) [6]. Importantly, it has been shown that *Pa* exposure can impair the efficacy of CFTRm in rescuing p.Phe508del-CFTR in primary human airway epithelial cells [7–10]. Given that many pwCF are already chronically colonized with Pa when the treatment with CFTRm started, chronic infection and inflammation might also explain the variable response to ETI. It is important to identify biological markers that can serve as reporters of the inflammatory status of airway mucosa in CF patients and the relationship with the recovery of CFTR function with CFTRm. We considered two proteins as possible markers: ATP12A and NFKBIZ.

ATP12A is a proton-potassium pump expressed in the apical membrane of the airway epithelium, whose role is considered detrimental in CF since its activity of proton secretion is not balanced by bicarbonate secretion through CFTR, thus causing abnormal acidification of the airway surface that impairs antimicrobial response [11, 12]. In cultured human bronchial epithelial cells, infectious and inflammatory stimuli have been reported to enhance ATP12A expression, such as bacterial supernatants, IL-4, IL-13, IL-1β, IL-6, IFN-γ, IL-17A alone or in combination with TNF-α [13–15]. Moreover, ATP12A is upregulated in CF human bronchi in respect to healthy controls [13], thus suggesting that its increase is probably linked to the infected and inflamed environment of CF airways. ATP12A is preferentially expressed in mucus-producing non-ciliated cells but, under pro-inflammatory stimuli, it can appear in ciliated cells as a sign of cell trans-differentiation [13, 15].

The NFKBIZ gene encodes for IkBζ protein, which belongs to the IkappaB protein family. This protein is expressed in bronchial epithelial cells and localized in the nucleus where it controls NF-kB transcriptional activity. NFKBIZ is induced by different inflammatory signaling pathways, including IL-1/TLR, IL-17 and JAK-STAT pathways [16, 17]. Stimulation by IL-1β, IL-17A and TNF-α has been shown to stabilize IkBζ mRNA and significantly prolong the NFKBIZ half-life [17–19]. Moreover, NFKBIZ upregulation has been reported in contexts of infection, kidney injury, metabolic diseases and hypoxic damage [17]. Although direct investigations into the role of NFKBIZ in CF are limited, its known functions in modulating NF-κB–mediated transcription and inflammatory gene expression indicate that it may contribute to the regulation of airway inflammation in CF. Therefore, in the current study we extended the investigation on the role of both ATP12A and NFKBIZ in the pathophysiological process in CF in airway epithelial cells.

## Material and methods

### Samples collection procedure

This is a prospective single center study. Data were collected at the CF Regional Reference Center of Florence, Italy (Meyer Children's Hospital IRCCS), a pediatric and adult center. The study included pwCF on ETI therapy, with either a homozygous F508del genotype or a heterozygous for F508del variant and one minimal function allele, identified through *CFTR* gene sequencing. The study was approved by the Ethical Committee of the CF Center (Florence, Ethics Clearance number 298/2021 and 104/2024). Informed consent was obtained during routine hospital visits from all enrolled patients and children's parents (or legal guardians).

Subjects with pulmonary exacerbation or acute rhinitis at the time of sampling were excluded according to [20]. The measurement of microbiological analysis, percent predicted forced expiratory volume (ppFEV1%), sweat chloride concentration was conducted in Florence using a procedure that has been recommended by international guidelines [21]. Blood samples, microbiological cultures from the nasal mucosa and nasal brushings were collected. Cells of the nasal mucosa were obtained from both nostrils using the Endobrush® cytological sampling brush (Biogyn, Mirandola, Italy), following previously described protocols [15, 22]. Each brush was immediately placed in a 15 mL tube containing either 10% neutral buffered formalin (05-01005Q; Bio-Optica, Milan, Italy) for fixation or culture medium (DMEM-F12) for live cell transport. Samples were then shipped to the laboratory. Cells were processed within 24 h after collection. These cells, referred to as “fresh primary nasal cells”.

To investigate the effect of anti-inflammatory drug, Dimethyl Fumarate (DMF), nasal epithelial cells were cultured for 16–18 days in air–liquid interface (ALI) as previously described [23, 24]. These HNE cultures were obtained by nasal cells isolated from brushing at Bambino Gesù Hospital (Rome, Italy). The collection and use of human airway epithelial cells for scientific research was approved by the Ethical Committee of Bambino Gesù Hospital (2961/2022).

### Immunofluorescence of nasal samples

The cytological brushes carrying fixed cells were processed as previously described [15, 22]. Briefly, the brush was transferred to a tube containing phosphate-buffered saline (PBS) and the cells were detached by passing the brush through a 200 µL micropipette tip (with the end cut off). After centrifugation (1000 rpm, 5 min), the cells were resuspended in a small volume of PBS and deposited on silanized glass slides (S4651-72EA, Sigma-Aldrich) placed in a humidified histological chamber. After 2–3 h, the samples were processed for immunofluorescence staining.

Cells underwent antigen retrieval with 10 mM citrate buffer, followed by permeabilization with 0.3% Triton X-100 in PBS for 5 min, block with 1% BSA in PBS (2 h), and incubation over-night at 4 °C with primary antibodies diluted in PBS containing 1% BSA (A7030, Sigma-Aldrich). Slides were stained with two sets of triple antibody combinations to detect ATP12A and NFKBIZ expression along with specific cell markers: 1. rabbit anti-ATP12A (HPA039526, Sigma-Aldrich), 1:400; mouse IgG1 anti-MUC5AC as marker of mucus-producing cells (MA5-12,178; Thermo Fisher Scientific), 1:200; mouse IgG2B anti–acetylated tubulin as marker of ciliated cells (T7451; MilliporeSigma), 1:300. 2. rabbit anti-NFKBIZ (IkB-zeta; 9244S; Cell Signaling) 1:200, combined with the same antibodies for mucus-producing and ciliated cells.

Then, samples were washed three times in PBS and incubated for 1 h in the dark with the appropriate combination of secondary antibodies diluted in PBS-BSA 1%: goat anti–rabbit Alexa Fluor 488 (A11008; Thermo Fisher Scientific) 1:200, goat anti–mouse IgG1 Alexa Fluor 546 (A2112; Thermo Fisher Scientific) 1:200, and goat anti–mouse IgG2B Alexa Fluor 633 (A21146; Thermo Fisher Scientific). After three additional PBS washes, slides were mounted using Fluoroshield with DAPI (MilliporeSigma) to stain nuclei.

Images were acquired with a laser scanning confocal microscope (TCS SPE; Leica Microsystems). For each sample, 200–500 cells were analyzed. ATP12A expression was quantified using previously described methods [15]. Two regions of interest (ROIs) were selected: one on the apical membrane (AM) and one in the cytoplasm (C), midway between the AM and the nucleus. The mean fluorescence intensity of each ROI was measured using ImageJ software (NIH), and only cells with an AM/C ratio greater than 2 were considered positive for ATP12A. This threshold was previously chosen to reflect the specific localization of ATP12A in the apical membrane [15]. To analyze NFKBIZ expression, which is localized to the nucleus, two circular ROIs were selected: one within the nucleus (N) and one in the cytoplasm (C). The mean fluorescence intensity of each ROI was calculated using ImageJ, and the N/C ratio was reported in the graphs.

### RNA isolation from nasal samples and quantitative reverse transcription PCR (RT-qPCR)

The cytological brushes carrying live cells in culture medium were processed by passing the brush through a 200 µL micropipette tip (with the tip cut off). The detached cells were centrifuged (1000 rpm, 5 min), then the pellet was lysed and the RNA was extracted using the reagents and protocols provided by RNeasy Mini Kit, 74,106, QIAGEN. Total RNA was converted to cDNA with an iSCRIPT cDNA synthesis kit (Biorad, Hercules, CA, USA) and quantitative real-time PCR was performed using Ssfast EvaGreen (Biorad, Hercules, CA, USA) and normalized to GAPDH [25]. The primer sets used for amplification were previously published [26, 27].

Nasal epithelial cells were seeded on 24-transwell (6.5 mm diameter, 0.4 μm pore size, Corning) and cultured at 37 °C for 18 days. Cells were treated at the basolateral side with 0.1% DMSO, or 50 μM Dimethyl Fumarate (DMF) in the presence of vehicle (LB) or clinical exoproducts of P. aeruginosa (EXO) isolated from pwCF for 24 h [9, 28]. Following treatment, cells were lysed and RNA was extracted as mentioned above.

### Statistical analysis

All data are represented as mean ± SD or SEM (as reported) of at least three independent replicates. GraphPad 9.0 software (San Diego, CA, USA) was used for all statistical analysis. Paired two-tailed t-test or one-way/two-way ANOVA were conducted as appropriate with a significance level *p* < 0.05. Data with multiple comparison were assessed using Tukey’s multiple comparison test with α = 0.05.

## Results

### Effects of ETI after 3 months in pwCF

**Fig. 1:**
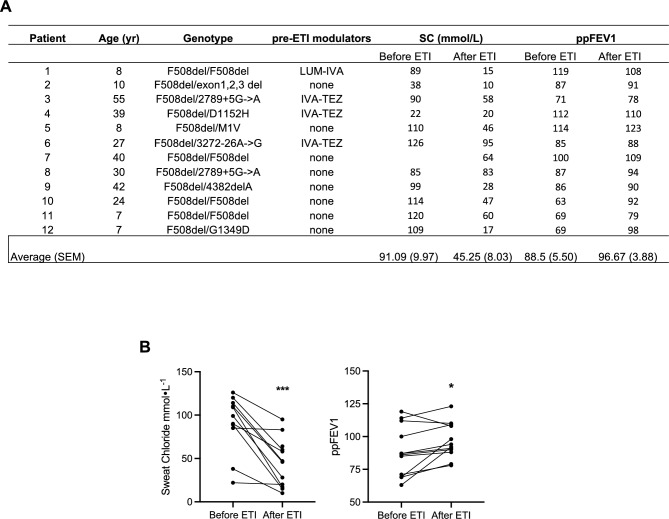
Effect of Elezacaftor/Tezacaftor/Ivacaftor (ETI) after 3 months in pwCF. **A** All 12 people with CF are represented by numbers, which correspond to data points throughout the text and figures. For each patients age, genotype, previous pharmacological treatment, sweat chloride (SC), and predicted forced expiratory volume in 1 s (ppFEV1: %) were indicated; **B** Graphs show the clinical data (sweat chloride and ppFEV1) before and after 3 months of ETI (elexacaftor/tezacaftor/ivacaftor) treatment in 12 CF patients. *p < 0.05; ***p < 0.001

We aimed to investigate the effects of Elexacaftor/Tezacaftor/Ivacaftor (ETI) in pwCF after 3 months of treatment. Primary nasal epithelial cells were collected from each patient by brushing both before and after ETI treatment. These cells (hereafter referred to as 'fresh primary nasal cells'), were processed directly for experimental studies without prior culture under air–liquid interface (ALI) conditions. Clinical characteristics of the selected cohort, including CFTR genotype and microbiology, are summarized in Fig. 1A. Clinical data on the percent predicted forced expiratory volume (ppFEV1%) and sweat chloride levels show that following 3 months of ETI treatment ppFEV1% was significantly increased by 8.17% while sweat chloride levels were significantly reduced (mean reduction: 45,84 mmol/L) (Fig. 1A, B).

**Fig. 2:**
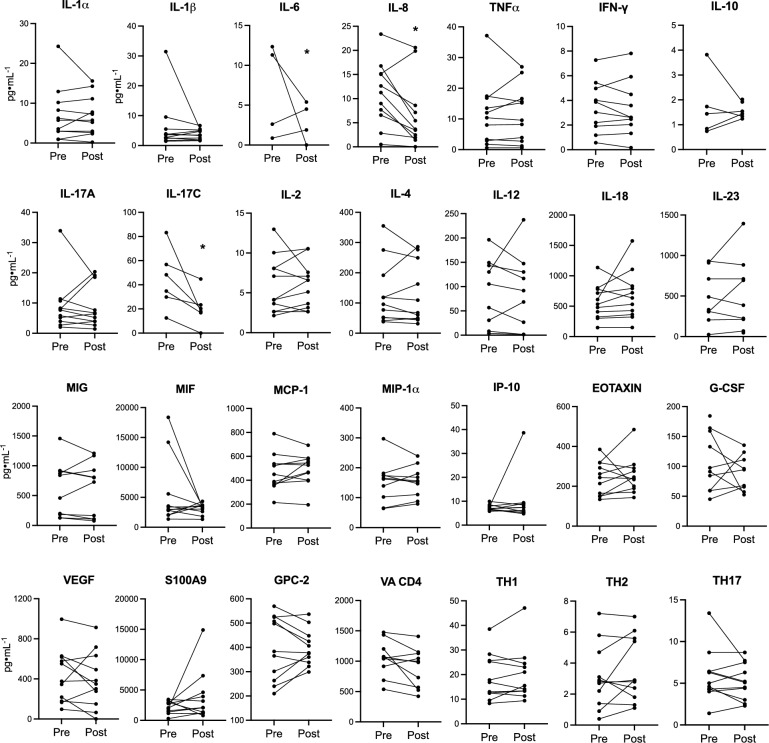
ETI treatment significantly reduces the IL-6, IL-8 and IL-17C levels in the serum of pwCF and modulates circulating T helper cells: Th1, Th2, and Th17 subsets. Bar graphs shown the mean ± S.D. of released cytokines in serum collected pre and 3 months post ETI treatment from 12 pwCF. (n = 3 technical replicates for each patient). Th1, Th2, and Th17 populations were analyzed by flow cytometry in PBMCs (peripheral blood mononuclear cells) and defined as CD4⁺CXCR3⁺CCR6⁻, CD4⁺CXCR3⁻CCR6⁻CCR4⁺, and CD4⁺CXCR3⁻CCR6⁺CD161⁺, respectively. *p < 0.05

**Fig. 3:**
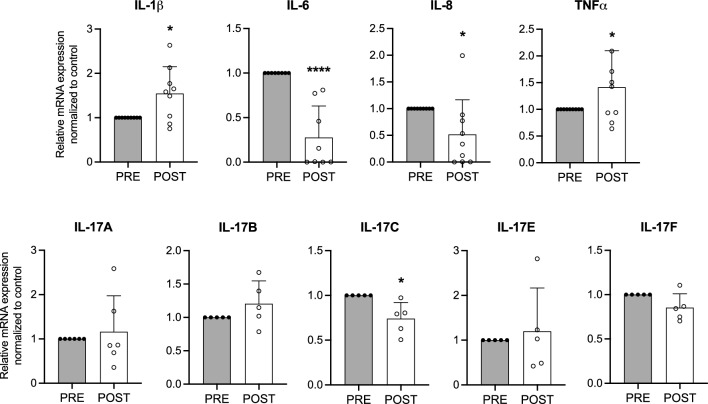
ETI treatment significantly reduces the IL-6, IL-8 and IL-17C mRNA levels in fresh primary nasal cells from pwCF. Bar graphs shown the mean ± SEM of Interleukin (IL)-1β, IL-6, IL-8, TNF-α, IL-17A, IL-17B, IL-17C, IL-17E, IL-17F mRNA levels in fresh primary nasal cells collected from 9 pwCF before (PRE) and after (POST) 3 months ETI treatment. (n = 3 technical replicates for each patient). *p < 0.05; ***p < 0.001

To assess ETI’s impact on inflammation, we measured pro- and anti-inflammatory markers levels in the serum of 12 pwCF before and after 3 months of ETI treatment. Interestingly, we observed a significant reduction of IL-6, IL-8 and IL-17C but no significant changes in other cytokines (i.e. IL-1β, TNF-α, INFγ, IL-4) (Fig. 2). In parallel, we analyzed the IL-1β, IL-6, IL-8, TNF-α, IL-17A, IL-17B, IL-17C, IL-17E and IL-17F mRNA levels in fresh primary nasal cells collected from 9 pwCF showing a significant reduction of IL-6, IL-8 and IL-17C expression levels (Fig. 3). Unexpectedly, we also observed significantly higher mRNA levels of IL-1β and TNF-α post-ETI treatment in these cells.

### Impact of ETI and dimethyl fumarate (DMF) on ATP12A levels in airway epithelial cells

**Fig. 4:**
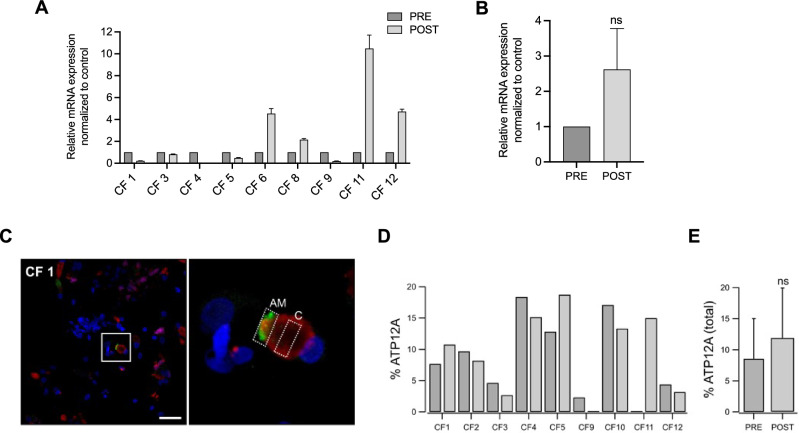
**ATP12A** expression did not change after three months of ETI treatment in fresh primary nasal cells from pwCF. **A**, **B** Bar graphs shown the mean ± SEM of ATP12A mRNA levels in fresh primary nasal cells from 9 pwCF before (PRE) and after (POST) 3 months ETI treatment.. **C** Representative confocal microscopy images at low (left) and high (right) magnification showing ATP12A protein localization (green) in freshly fixed nasal brushing samples from CF individuals. Co-staining includes MUC5AC (red) and acetylated tubulin (magenta), marking mucus and cilia, respectively. The box in the low-magnification panels highlight the region shown at higher magnification. The regions of interest (ROIs) outlined in the high-magnification images were used for quantifying ATP12A expression (AM = apical membrane; C = cytosol). Scale bar: 25 μm. **D** Bar graph showing the percentage of ATP12A-positive cells in individual CF patients before (dark gray) and after (light gray) ETI treatment **E** Bar graph summarizing the percentage of ATP12A-positive cells in all 9 patients before and after ETI treatment (ns = not significant Student’s t-test)

Next, we investigated the impact of three months of ETI treatment on ATP12A levels in fresh primary nasal cells. Interestingly, we observed a reduction in ATP12A mRNA levels in 4 out of 9 pwCF (CF1, CF4, CF5, CF9) after ETI treatment (Fig. 4A). On the other hand, there was no change (e.g., CF 3) or an increase in ATP12A mRNA levels in the remaining 4 out of 9 pwCF (Fig. 4A). Overall, there was an increase in the mean mRNA levels of ATP12A in fresh primary nasal cells after ETI treatment, but it did not reach statistical significance (Fig. 4B).

To quantify the number of cells expressing ATP12A, we performed immunofluorescence studies (Fig. 4C). We observed variability in the change of ATP12A-positive cells in pwCF before and after ETI treatment among individuals. Some of them (CF3, CF4, CF9, CF11) showed the same trend (increase/decrease) of ATP12A mRNA and protein levels after ETI treatment. Similarly to mRNA data, the mean of ATP12A-positive cells after ETI treatment seems to increase, but this did not reach statistically significance (Fig. 4E).

**Fig. 5:**
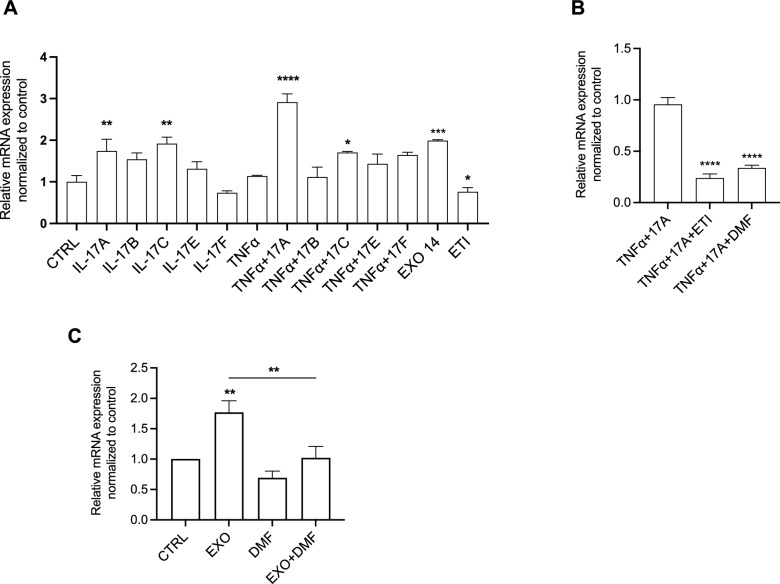
DMF, an anti-inflammatory drug, decreased the ATP12A levels in airway epithelial cells. **A** Bar graphs shown the mean ± SEM of ATP12A mRNA levels in F508del-CFTR CFBE cells treated with 20 ng/mL IL-17A, 10 ng/mL IL-17B, 50 ng/mL IL-17C, 50 ng/mL IL-17E, 50 ng/mL IL-17F, 10 ng/mL TNF-α, 1% EXO14 and 3 μM VX-445 + 3 μM VX-661 + 1 μM VX-770 for 24 h at 37 °C. **B** Bar graphs shown the mean ± SEM of ATP12A mRNA levels in F508del-CFTR CFBE cells treated with 20 ng/mL IL-17A + 10 ng/mL TNFα ± 3 μM VX-445 + 3 μM VX-661 + 1 μM VX-770 ± 50 μM DMF for 24 h at 37 °C. **C** Bar graphs shown the mean ± SEM of ATP12A mRNA levels in primary nasal cells from 6 pwCF (2 homozygous for F508del mutation and 4 heterozygous) after stimulation with lysogeny broth (CTRL), clinical exoproducts of P. aeruginosa (EXO) isolated from the corresponding donor treated ± 50 μM Dimethyl Fumarate (DMF) for 24 h at 37 °C. (n = 3 technical replicates for each patient). *p < 0.05; **p < 0.01; ***p < 0.001; ****p < 0.0001

Recently, we demonstrated a relevant role of IL-17 cytokines in the pathophysiology of CF [28]. To assess the impact of the IL-17 family on ATP12A expression, F508del-CFTR CFBE cells were treated with IL-17A, IL-17B, IL-17C, IL-17E, IL-17F, and TNF-α. In addition, to mimic chronic infection with *P. aeruginosa*, we treated the F508del-CFTR cells with exoproducts (EXO) from clinical strains of *P. aeruginosa* isolated from the bronchoalveolar lavage (BAL) of one pwCF heterozygous for the F508del mutation. Interestingly, we found a significant increase in ATP12A mRNA levels following treatment with IL-17A, IL-17C, and their combination with TNF-α and EXO infection, while a significant decrease was observed after ETI exposure (Fig. 5A). Next, we investigated the effect of ETI and DMF on F508del-CFTR CFBE cells under inflammatory conditions induced by the combination of IL-17A and TNF-α. We observed a significant reduction of IL-17A + TNF-α-induced ATP12A levels after ETI and DMF treatment (Fig. 5B).

Finally, we investigated whether the effect of EXO infection on ATP12A levels observed in CFBE cells also occurred in primary nasal epithelial cells exposed to EXO isolated from the *P. aeruginosa* strain from the corresponding pwCF. Patient-derived cultures were generated from nasal brushings obtained from 6 pwCF (2 homozygous for the F508del mutation and 4 pwCF heterozygous from whom the *P. aeruginosa* exoproducts were isolated). A significant increase in ATP12A expression levels was observed after EXO stimulation, which was significantly reduced by DMF treatment (Fig. 5C).

### NFKBIZ expression is upregulated under inflammation response and down regulated after DMF treatment in airway epithelial cells

**Fig. 6:**
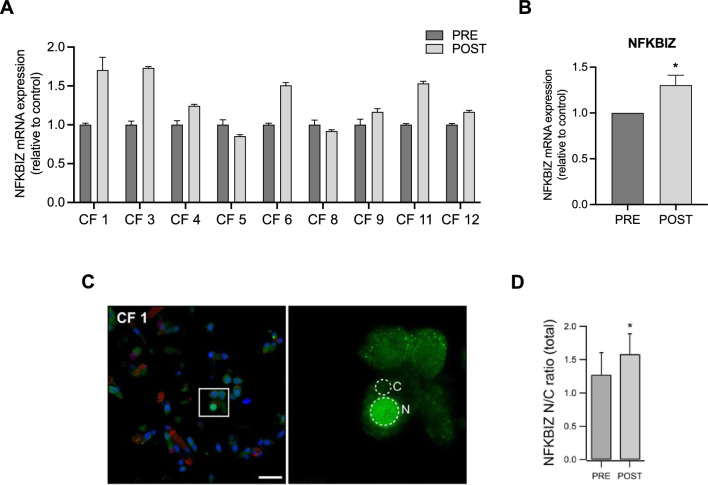
NFKBIZ expression is increased after three months of ETI treatment in fresh primary nasal cells from pwCF. (**A**, **B**) Bar graphs shown the mean ± SEM of NFKBIZ mRNA levels in fresh primary nasal cells from 9 pwCF before (PRE) and after (POST) 3 months ETI treatment. **C** Representative confocal microscopy images at low (left) and high (right) magnification showing NFKBIZ protein localization (green) in freshly fixed nasal brushing samples from CF individuals. Co-staining includes MUC5AC (red) and acetylated tubulin (magenta), marking mucus and cilia, respectively. The box in the low-magnification panels highlight the region shown at higher magnification. The regions of interest (ROIs) outlined in the high-magnification images were used for quantifying NFKBIZ expression (N = nucleus; C = cytosol). Scale bar: 25 μm. **D** Bar graph summarizing the NFKBIZ N/C ratio in all 9 patients before and after ETI treatment (*p < 0.05; Student’s *t*-test)

The next step was to investigate NFKBIZ expression in fresh primary nasal cells from nine pwCF both before and after three months of ETI treatment. Despite patient-to-patient variability, we observed an overall significant upregulation of NFKBIZ mRNA levels after ETI treatment (Fig. 6A, B). Immunofluorescence analysis showed a predominant nuclear localization of the NFKBIZ protein, which was also significantly higher in fresh nasal cells after three months of ETI treatment (Fig. 6C, D).

**Fig. 7:**
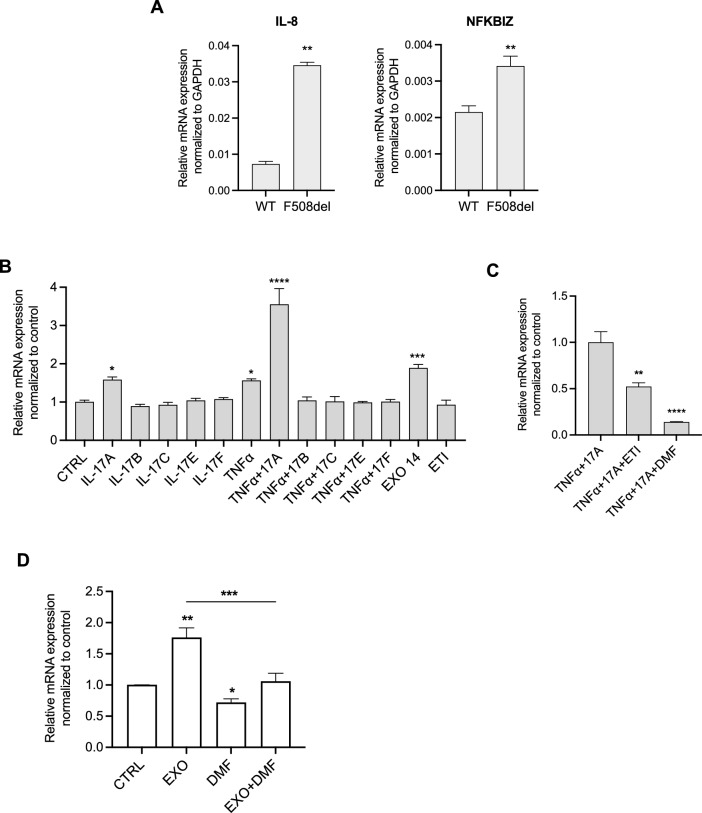
NFKBIZ expression is significantly decreased by DMF and ETI treatment in F508del-CFTR CFBE and primary nasal epithelial cells. **A** Bar graphs show the mean ± SEM of IL-8 and NFKBIZ levels in wild-type (WT) and F508del-CFTR CFBE cells (n = 3). **B** Bar graphs shown the mean ± SEM of NFKBIZ mRNA levels in F508del-CFTR CFBE cells treated with 20 ng/mL IL-17A, 10 ng/mL IL-17B, 50 ng/mL IL-17C, 50 ng/mL IL-17E, 50 ng/mL IL-17F, 10 ng/mL TNF-α, 1% EXO14 and 3 μM VX-445 + 3 μM VX-661 + 1 μM VX-770 for 24 h at 37 °C. **C** Bar graphs shown the mean ± SEM of NFKBIZ mRNA levels in F508del-CFTR CFBE cells treated with 20 ng/mL IL-17A + 10 ng/mL TNF-α ± 3 μM VX-445 + 3 μM VX-661 + 1 μM VX-770 ± 50 μM DMF for 24 h at 37 °C. **H** Bar graphs shown the mean ± SEM of NFKBIZ mRNA levels in primary nasal cells from 6 pwCF (2 homozygous for F508del mutation and 4 heterozygous) after stimulation with lysogeny broth (CTRL), clinical exoproducts of *P. aeruginosa* (EXO) isolated from the corresponding donor treated ± 50 μM Dimethyl Fumarate (DMF) for 24 h at 37 °C. (n = 3 technical replicates for each patient). *p < 0.05; **p < 0.01; ***p < 0.001; ****p < 0.0001

Before investigating the impact of inflammation and infection on NFKBIZ expression in primary nasal cells, we measured basal NFKBIZ mRNA levels in CFBE cells. Interestingly, similar to IL-8, we found a significant NFKBIZ upregulation in F508del-CFTR compared to WT-CFTR CFBE cells (Fig. 7A). We then treated F508del-CFTR CFBE cells with IL-17A, IL-17B, IL-17C, IL-17E, IL-17F, TNF-α, EXO from one pwCF heterozygous for the F508del mutation, and ETI. Interestingly, we found a significant increase in NFKBIZ mRNA levels following treatment with IL-17A, TNF-α, their combination, and EXO infection (Fig. 7B). Notably, ETI treatment alone did not alter the basal NFKBIZ levels in F508del-CFTR CFBE cells. However, as shown in Fig. 7C, NFKBIZ levels induced by IL-17A + TNF-α were significantly reduced following treatment with ETI or DMF.

Finally, we investigated NFKBIZ expression in primary nasal cells from 6 pwCF after infection with clinical EXO of *P. aeruginosa* isolated from the corresponding patient. We demonstrated a significant increase in NFKBIZ mRNA levels after EXO stimulation, which was reduced after DMF treatment (Fig. 7D).

## Discussion

Restoration of CFTR function by the triple combination CFTRm with ETI has been shown to have the downstream effect of reduction of chronic airway inflammation, as assessed either in sputum or plasma of pwCF. However, it's important to note that inflammatory marker levels did not fully return to those observed in healthy subjects [4, 29]. In this study, we evaluated 28 inflammatory protein markers in the plasma of pwCF before and after three months of ETI treatment. Consistent with previous reports by others [4, 29], we found a significant reduction of the neutrophil chemokine IL-8 and of the pro-inflammatory cytokine IL-6 (Fig. 2), whereas no significant changes were appreciated in the plasma concentration of the other major pro-inflammatory cytokines IL-1β and TNF-α [4, 29, 30]. Interestingly, our study uniquely identified a significant reduction in plasma concentration of IL-17C, a pro-inflammatory cytokine which is released not only by immune cells but also by the respiratory epithelial cells upon different pro-inflammatory challenges [28], a cytokine that is gaining increasing interest in CF lung disease, both on pathophysiological aspects and possibly for its application as a marker to monitor changes of the inflammatory status.

Considering that biological samples collected in the conductive airway tract from pwCF could be more reliable than plasma to detect variation of inflammation in CF chronic lung disease, it's important to acknowledge the challenge of sputum collection in ETI-treated pwCF due to dramatic reduction in sputum volume. As an alternative approach, we explored the utility of nasal brushing to collect surface cells from the nasal mucosa. This method can provide valuable information on both defective CFTR correction and inflammatory status on an individual basis [9]. We thus investigated the variation of transcription of the four paradigmatic genes IL-8, IL-6, IL-1β and TNF-α, together with five IL-17 isoforms that we previously found expressed in these cells [28]. Notably, steady state transcript levels of IL-8, IL-6 and IL-17C genes are significantly decreased after 3 months of ETI (Fig. 3), consistently with their variation in concentrations observed in the plasma of the same pwCF (Fig. 2). Surprisingly, as regards the results reported in sputum by others [29, 31], we found incremental steady state levels of IL-1β and TNF-α mRNA in freshly collected nasal cells of pwCF after three months of ETI (Fig. 3). This discrepancy may have been explained as nasal epithelial cells reflect only one selected portion of the airways, whereas sputum reports more closely the overall condition of the bronchial tree. However, reduction of IL-1β mRNA in the epithelial component of nasal cells collected by brush 3 months after ETI treatment was observed by others [31], by RNAseq analysis. Thus, an alternative possibility is that some of the nasal brush samplings post ETI treatment have been carried out in the presence of an undetectable upper respiratory infection in its initial phase, either bacterial or viral, which upmodulates the expression of the classical acute early-phase mediators of inflammation, namely IL-1β and TNF-α. As a matter of fact, CFTRm ETI has been reported to decrease the bacterial infection and to change the microbiota of the airway tract in pwCF, but the recurrence of acute respiratory infections still persists after ETI treatment [32, 33]. As a limitation of our study, although we selected the pwCF under study presumed to be in a stable condition, we did not perform PCR analyses for respiratory viruses to exclude potential effects of an acute infection on inflammatory markers. Thus, the occurrence of a viral or bacterial pro-inflammatory challenge in the respiratory tract makes compatible the increase of IL-1β and TNF-α mRNA in freshly obtained nasal cells from some of the pwCF included in this study (Figure S1) and possibly reconciles the lack of reduction of these two cytokines in the plasma of pwCF post ETI treatment (Fig. 2). Thus, acknowledging that the recruitment of pwCF with complete absence of any environmental bacterial or viral infectious stimulus in the airway tract is a critical task, we should strongly underline that an initial airway infection in the days preceding the collection biological samples from our pwCF cannot be completely excluded and represents a major limitation of this investigation.

In our study, we investigated the expression of two proteins as possible markers of inflammation: ATP12A and NFKBIZ. In two previous studies, we found evidence of increased ATP12A expression in bronchi and freshly collected nasal epithelial cells from CF patients [13, 15]. This finding may be the result of an inflammatory milieu that promotes ATP12A gene transcription. In the present study, we confirmed that dependence of ATP12A expression on inflammatory stimuli. We compared ATP12A expression, at mRNA and protein levels, in nasal epithelial cells of CF patients, before and after ETI treatment. The results indicated a lack of ATP12A downregulation after treatment. This result may indicate that, despite CFTR rescue, there is still a persistent level of inflammation, at least in the nasal mucosa, possibly related to an initial respiratory infection condition at the time of biological sampling of the pwCF recruited in this study.

The NFKBIZ gene encodes for IkBζ protein, an unconventional IkB protein that functions as a cofactor for the transcription factor NF-kB [17]. NFKBIZ gene expression is induced by different pro-inflammatory pathways, namely MyD88-dependent signals, like those downstream IL-1 receptor (IL1R) and microorganism-activated Toll-Like Receptors (TLRs) 1, 2, 4 and 5, together with IL-17 receptors [17]. In addition, NFKBIZ mRNA was shown to be stabilized by IL-1β, IL-17A and TNF-α, which significantly prolong the NFKBIZ half-life signal downstream IL1R and TLRs [17–19, 34, 35]. Therefore, our finding that average NFKBIZ mRNA is increased in samples of fresh primary nasal cells isolated from pwCF (Fig. 5) is consistent with the average IL-1β and TNF-α mRNA levels observed in the same samples (Fig. 3). In this respect, although multiple pathways are involved in NFKBIZ mRNA induction and stabilization, it is interesting to observe a significant correlation between the levels of IL-1β and TNF-α mRNA on one side and those of NFKBIZ mRNA on the other side (Figure S2), a correlation of NFKBIZ mRNA which is not significantly evident for IL-8 or IL-6 mRNA levels. The pattern of induction of NFKBIZ expression has been also verified in the F508del-CFTR CFBE bronchial epithelial cell line, where we confirmed the role of IL-17A, TNF-α and bacterial exoproducts (EXO), where the combined stimulation with TNF-α and IL-17A provides the strongest activation (Fig. 6B). The results obtained in fresh nasal primary cells and in F508del-CFTR CFBE bronchial cells confirm that overexpression of NFKBIZ mRNA is associated with pro-inflammatory soluble mediators or bacterial challenge in respiratory epithelial cells (Figs. 5 and 6B). This could also explain the discrepancy of the effect of ETI on NFKBIZ mRNA expression between freshly-isolated nasal epithelial cells (Fig. 5) and nasal epithelial cells kept in culture for weeks up to ALI differentiation (Fig. 6C), since the latter cells, that were maintained under sterile conditions in vitro, could more clearly show the effect of ETI after a well-defined pro-inflammatory challenge, such as TNF-α and IL-17. Interestingly, dimethyl fumarate (DMF) strongly decreases the NFKBIZ mRNA levels induced in vitro either by the association of TNF-α and IL-17A or by bacterial exoproducts (EXO), both in F508del-CFTR CFBE bronchial cells and in fully differentiated primary nasal epithelial cells in culture (Fig. 6C andD), suggesting the feasibility of targeting the excessive NFKBIZ expression with anti-inflammatory and/or anti-oxidant molecules [26]. In this respect, it has been shown that ETI reduces but does not stop inflammation in pwCF [29–33], thus anti-inflammatory drugs associated with Highly Effective Modulator Therapy (HEMT) is recommended [20, 21, 36]. As daily use of current anti-inflammatory drugs such as ibuprofen results in severe adverse effects in pwCF, the discovery of novel safe and effective anti-inflammatory molecules is an unmet need [36]. Different inhibitors of proteases are in trial to mitigate the adverse effects of the effectors of tissue damage released by the excess of activated neutrophils in the lumen of CF conductive airways [37]. DMF, as anti-oxidant molecule could mitigate the excessive Reactive Oxygen Species released by neutrophils whereas in parallel it reduces pro-inflammatory cytokines and neutrophil chemokines [26]. Paying attention that each anti-inflammatory molecule under development does not negatively interfere with the rescue of mutated CFTR [38] and that the reduction of inflammation will not blunt immune defenses, resulting in critical adverse effects with bacterial overgrowth [39], discovery of safe and effective novel anti-inflammatory drugs is a relevant field of investigation for the cure of pwCF. A basal pro-inflammatory status in respiratory epithelial cells from pwCF carrying mutant CFTR protein has been shown since long time, in particular in cells carrying the F508del CFTR allele [6]. Here we confirm that expression of IL-8 gene, a hallmark of CF chronic lung disease that drives excessive recruitment of neutrophils into CF conductive airways [40], is significantly increased in F508del-CFTR CFBE bronchial cells (Fig. 6A). Interestingly, also NFKBIZ mRNA is significantly higher in F508del-CFTR cells in respect to the cells bearing wild type CFTR, in the absence of proinflammatory challenges (Fig. 6A). Correction of the basic defect of the mutated CFTR protein with the triple combination CFTRm ETI, which partially reduces lung inflammation in pwCF [4, 29], was found by others to reduce the expression of IL-8 and IL-6 induced by the pro-inflammatory cytokine TNF-α in bronchial cells [41], whereas we previously found that ETI reduces IL-8, IL-6, IL-1β and TNF-α after exposure to bacterial exoproduct in nasal primary cells [9]. Intriguingly, we previously found that ETI was found ineffective on IL-8 and IL-6 when nasal primary cells were bearing CFTR mutations that are ETI-resistant to the functional rescue of the CFTR-mediated ion transport [41]. Here we found that also NFKBIZ is reduced by ETI in F508del-CFTR CFBE bronchial cells when its expression is upregulated by a pro-inflammatory challenge (e.g. TNF-α + IL-17A) (Fig. 6C) but not under basal conditions (Fig. 6B). Considering collectively the previous and present findings, it could be possible to speculate that the signaling regulation of expression of NFKBIZ is closely associated with that of other pro-inflammatory genes overexpressed both at constitutive and stimulated conditions in CF respiratory epithelial cells (e.g. IL-8). Why ETI is able to significantly reduce the expression of ATP12A and NFKBIZ mRNA after induction with TNF-α and IL-17 in F508del-CFTR CFBE cells in vitro (Figs. 4 and 6) is an interesting finding that further strengthens a general anti-inflammatory role of ETI besides its main action on the rescue of F508del-CFTR. Although ETI is known to reduce in parallel to ATP12A and NFKBIZ mRNA also other inflammatory genes, we cannot simply causatively correlate these co-modulations of expression of genes involved in inflammation, thus the answer to this interesting question will require future investigation.

Although we showed here that expression of NFKBIZ gene in nasal or bronchial CF respiratory epithelial cells is often associated with that of other pro-inflammatory genes (e.g. IL-8), its role in CF lung disease remains so far elusive. However, IkBζ protein has been shown to play specific roles in the pathophysiology of other diseases such as psoriasis, chronic enteritis, alcoholic hepatitis, rheumatoid arthritis [17]. For instance, NFKBIZ was found overexpressed in liver samples of subjects affected by alcohol-associated liver disease, in significant correlation with proinflammatory genes like TNF-α, ICAM, VCAM and CXCL1 [42]. In this pathology, IkBζ protein was found to play a key non redundant role in activating neutrophil chemotaxis in liver tissue, where hepatocyte-specific NFKBIZ-deficient mice showed a marked reduction of neutrophil infiltration in the liver upon alcoholic challenge [42]. Similarly in rheumatoid arthritis, stromal fibroblasts are pivoting inflammation by secreting pro-inflammatory cytokines like IL-6 and by recruiting neutrophils and monocytes by releasing chemokines such as CXCL1, CXCL2, CXCL3 and IL-8 upon induction with TNF-α and IL-17A. Interestingly, upon this combined cytokine stimulation, IkBζ was found to bind NF-kB p65 and p100/52 subunits acting as activator of IL-8 secretion, which was significantly reduced after silencing NFKBIZ gene expression [43]. Reminiscent of the adverse effect that the excessive neutrophil recruitment exerts on CF progressive lung tissue damage [40], the intriguing possibility is open that NFKBIZ expression might be relevant in CF neutrophil chemotaxis, a hypothesis that will require future investigation.

**Fig. 8:**
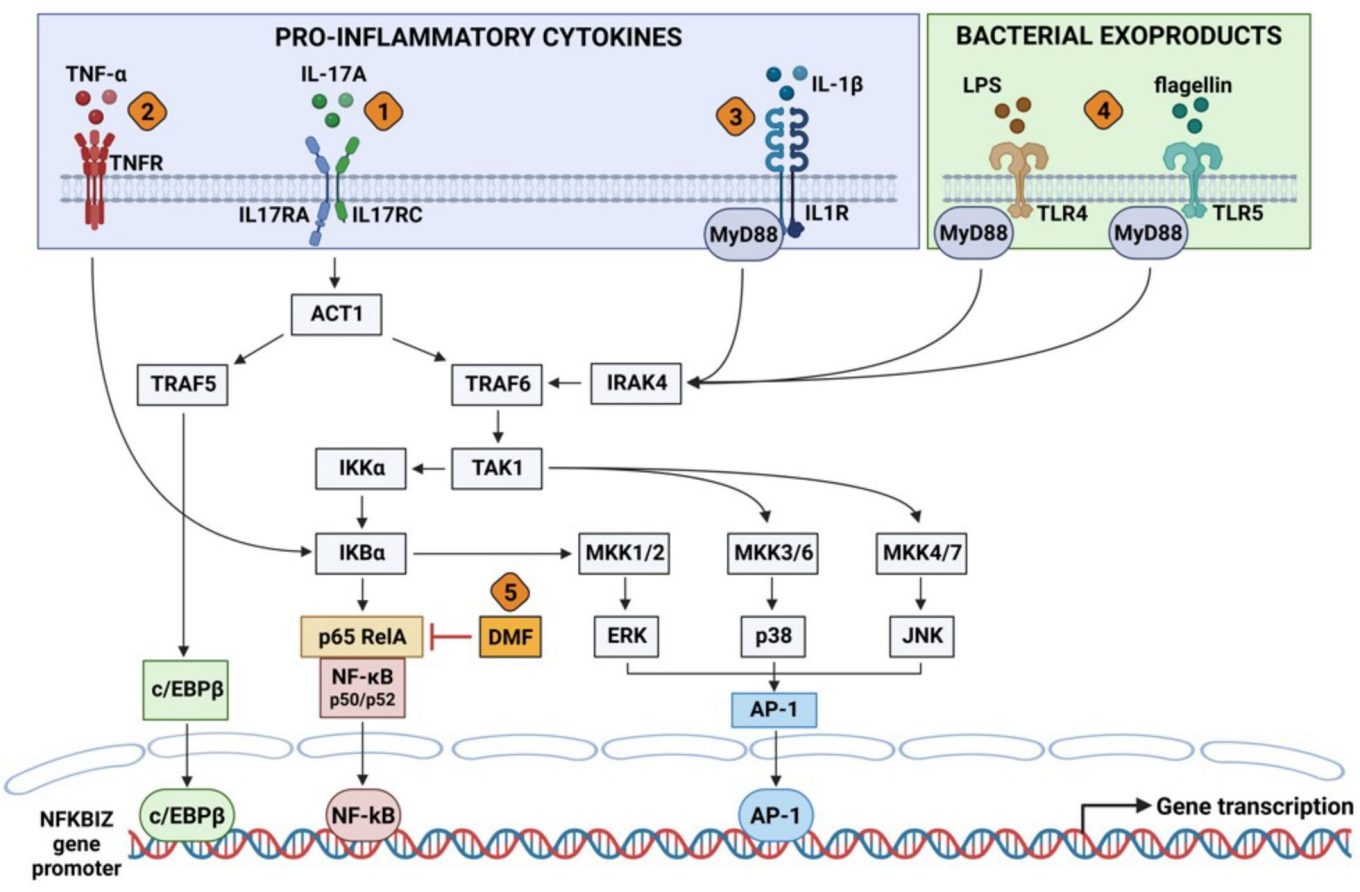
Proposed inflammatory pathways involved in NFKBIZ transcription and role of DMF in airway epithelial cell models. The figure summarizes the major intracellular signals known to be involved in NFKBIZ transcription upon IL-17A and TNF-α stimulation or proinflammatory challenge with bacterial exoproducts. Signaling pathways, as summarized in Feng et al. [17], are simplified and adapted to the respiratory epithelial model and results presented here. 1. Interaction of IL-17A with IL-17RA/C dimer receptor activating different major checkpoints of the Mitogen Activated Protein Kinases (MAPK) ERK, p38 and JNK module and the NF-kB module, leading to the activation and nuclear translocation of transcription factors c/EBPβ, NF-kB and AP-1. 2. Interaction of TNF-α with the trimeric TNFR activates NF-kB and part the MAPK module, inducing NFKBIZ transcription. Although IL-17A and TNF-α are separately able to activate NFKBIZ transcription, the combined addition of both cytokines produces a much stronger induction of activation, as reported in Fig. 6B. 3. Challenging respiratory epithelial cells with IL-17A is known to induce the expression and release of different cytokines and chemokines, such as IL-1β, IL-6, TNF-α, IL-4 and IL-13, IL-8, G-CSF, as Others and we previously reported [15, 28]. Thus, an autocrine amplification of the circuitry of inflammation through secreted TNF-α and IL-1β after IL-17 challenge is expected [15], helping in parallel the stabilization of the NFKBIZ transcript [17–19]. In addition, the secretion of mucins on the apical side of polarized epithelia, that increases the already exaggerated viscosity of CF airway surface fluid, is a relevant additional pro-inflammatory stimulus [15]. 4. In the classical CF condition of infection with different bacteria, including *Pseudomonas aeruginosa*, LPS and flagellin released from bacteria interact with Toll like Receptors (TLRs) 4 and 5, respectively. TLRs activate IRAK4 and TRAF6 through MyD88 adaptor protein and, downstream, the NF-kB and MAPK modules, inducing the transcription of a plethora of genes involved in inflammation, including NFKBIZ. 5. As shown in Fig. 6C and D, DMF inhibits NFKBIZ transcription stimulated either by TNF-α/IL-17A or by bacterial exoproducts. DMF is known to reduce pro-inflammatory genes by inhibiting the phosphorylation of p65 NF-kB, its nuclear entry and the NF-kB-DNA complex formation [44, 45], a multiple mechanism that could be also reasonably implied in the inhibition of NFKBIZ transcription

In conclusion, we demonstrated that NFKBIZ and ATP12A are upregulated by inflammatory and infective stimuli, and their expression is decreased by the anti-inflammatory drug DMF (Fig. 8). The lack of overall NFKBIZ and ATP12A downregulation after ETI in vivo may be explained with a persistence of inflammation. In this regard, we found previously that these two proteins are particularly upregulated by IL-17A [15]. Considering that in blood (Fig. 2) and nasal samples (Fig. 3) there was no decrease in IL-17A levels post-ETI, this could explain the sustained ATP12A and NFKBIZ expression.

## Supplementary Information

Below is the link to the electronic supplementary material.

Supplementary file1 (DOCX 176 KB)

## Data Availability

No datasets were generated or analysed during the current study.
