# Supplementary material for: Evaluation of ATP12A and NFKBIZ as potential markers of inflammatory status in cystic fibrosis airway epithelial cells
Source: Inflamm Res. 2026 Mar 17;75(1):51. doi: 10.1007/s00011-026-02210-z (PMC12995941; doi:10.1007/s00011-026-02210-z)
Supplement: Supplementary file 1 — Supplementary file1 (DOCX 176 KB) [file 11_2026_2210_MOESM1_ESM.docx]

Figure S1 Figure S1: ETI induced variable pro-inflammatory mRNA levels in in fresh primary nasal cells from pwCF. Total RNA was extracted from fresh primary nasal epithelial cells and qRT-PCR was performed in order to quantify Interleukin (IL)-1β, IL-6, IL-8 and TNF−α mRNA levels. Bar graphs show the mean (±SEM) of n =3 technical replicates for each donor.


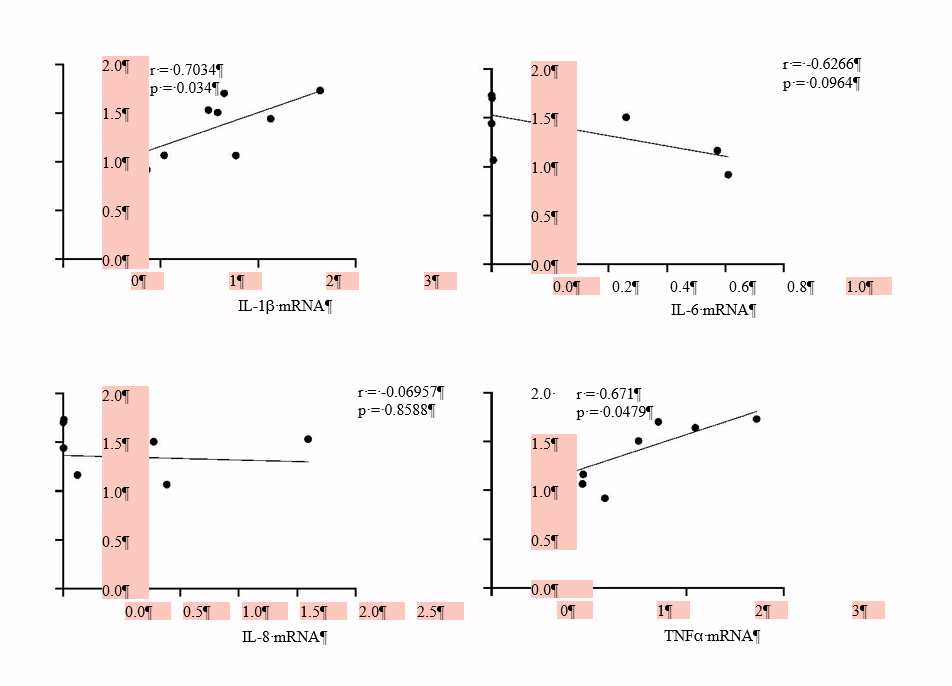


Figure S2: Correlation of NFKBIZ mRNA and IL-1β/TNF-α mRNA levels in in fresh primary nasal cells from pwCF. Correlation between the mRNA levels of NFKBIZ (expressed as ratio of POST/PRE NFKBIZ mRNA level) and pro-inflammatory cytokines (expressed as ratio of POST/PRE IL1β, IL-6, IL-8 and TNFα mRNA levels).
